# A Theory of Mental Frameworks

**DOI:** 10.3389/fpsyg.2023.1220664

**Published:** 2023-07-20

**Authors:** Tracey Tokuhama-Espinosa, Kristin Simmers, Danielle Batchelor, Allen Drew Nelson, Cynthia Borja

**Affiliations:** ^1^Harvard University Extension School, Faculty of Arts and Sciences, Cambridge, MA, United States; ^2^Connections: The Learning Sciences Platform, Quito, Ecuador; ^3^Neag School of Education, CT Institute for Brain and Cognitive Science University of Connecticut, Storrs, CT, United States; ^4^The Decision Lab, Independent Behavioral Science Research, Montreal, QC, Canada

**Keywords:** mental frameworks, problem-solving, critical thinking, learning how to learn, mind-brain-education, higher order cognitive functions, mental schemata

## Abstract

Problem-solving skills are highly valued in modern society and are often touted as core elements of school mission statements, desirable traits for job applicants, and as some of the most complex thinking that the brain is capable of executing. While learning to problem-solve is a goal of education, and many strategies, methodologies, and activities exist to help teachers guide the development of these skills, there are few formal curriculum structures or broader frameworks that guide teachers toward the achievement of this educational objective. Problem-solving skills have been called “higher order cognitive functions” in cognitive neuroscience as they involve multiple complex networks in the brain, rely on constant rehearsal, and often take years to form. Children of all ages employ problem solving, from a newborn seeking out food to children learning in school settings, or adults tackling real-world conflicts. These skills are usually considered the end product of a good education when in fact, in order to be developed they comprise an ongoing process of learning. “Ways of thinking” have been studied by philosophers and neuroscientists alike, to pinpoint cognitive preferences for problem solving approaches that develop from exposure to distinct models, derived from and resulting in certain heuristics used by learners. This new theory paper suggests a novel understanding of the brain’s approach to problem solving that structures existing problem-solving frameworks into an organized design. The authors surveyed problem-solving frameworks from business administration, design, engineering, philosophy, psychology, education, neuroscience and other learning sciences to assess their differences and similarities. This review lead to an appreciation that different problem-solving frameworks from different fields respond more or less accurately and efficiently depending on the kinds of problems being tackled, leading to our conclusion that a wider range of frameworks may help individuals approach more varied problems across fields, and that such frameworks can be organized in school curriculum. This paper proposes that explicit instruction of “mental frameworks” may help organize and formalize the instruction of thinking skills that underpin problem-solving–and by extension–that the more such models a person learns, the more tools they will have for future complex problem-solving. To begin, this paper explains the theoretical underpinnings of the mental frameworks concept, then explores some existing mental frameworks which are applicable to all age groups and subject areas. The paper concludes with a list of five limitations to this proposal and pairs them with counter-balancing benefits.

## 1. Introduction

Education has long been seen as a social equalizer in society (Bernardi and Ballarino, 2016), and educational goals regularly adapt to modern times (e.g., Bird and Bhardwaj, 2022) in terms of *what*, *how*, and even *why* things are taught (Darling-Hammond et al., 2020). While the specific content of the educational experience is regularly debated, a constant over the past few decades has been to emphasize *how* to think, not *what* to think, in order to encourage life-long learning (Velez and Power, 2020). Indeed, the formation of “deep thinkers” (Helm, 2015) and “deep learners” (Fullan et al., 2018) is perceived as a way of time-proofing educational content by learning a *process* to approach problems (Gunawardena and Wilson, 2021). Some argue that by helping students to learn to think for themselves, society is also helping its members develop literacies which may help protect one’s values, by shielding against invasions from undue influences on social media and so called “fake news” (Paul and Elder, 2019). Thinking skills are needed to resolve both simple and complex problems, as explicitly learning critical thinking skills improve problem-solving, just as rehearsal of problem-solving enhances overall thinking (Belecina and Ocampo, 2018). This “chicken and egg” relationship of thinking to problem solve (or problem-solving to think) has often led to these concepts being used interchangeably in the literature (e.g., Shanta and Wells, 2022). Problem-solving emerged as a desirable skillset in modern education about a 100 years ago when educators suggested that the ability to resolve problems outside of school subject areas displayed transfer and higher order thinking, which was also the responsibility of education (e.g., Dewey, 1930; Burton, 1929). Burton went so far as to call problem-solving one of five forms of learning, and Dewey noted that, “Education is not preparation for life; education is life itself” (Dewey, 1930, p. 267).

Current school trends designed to respond to the call for problem-solvers has led to the successful application of pedagogical approaches, such as problem-based learning (e.g., Uluçınar, 2023), inquiry-based practice (e.g., Öztürk et al., 2022), and personalized learning through technology (e.g., Wang et al., 2022). While such interventions tend to have a positive result on problem-solving skills, they are not organized, structured, nor presented as a single curriculum.

### 1.1. Problem statement

There are differing opinions about the best way to teach problem-solving skills in schools. Most of the literature offering evidence-based interventions surround techniques (e.g., Proctor, 2020), strategies (e.g., Zhao et al., 2019), methodologies (e.g., Casiraghi and Aragão, 2019), and activities for critical and creative thinking (e.g., Akgun and Sharma, 2023), showing a range of excellent approaches and spanning all age groups. However, there is less literature on how all these become habituated as thinking *processes* in the brain, which over time (starting in early childhood and consolidating in adulthood), become heuristic approaches to problem solving. We propose that the habituated heuristics of thinking explain the speed and agility of problem solving. Furthermore, we suggest that a person with many different possible mental frameworks to choose from in the problem-solving process is like the mechanic with an extensive tool box, or the painter with a broader pallet of colors, whereby more options enhance the likelihood of a good outcome. This exposure to different problem-solving approaches also explains why certain complex or dynamic problems may be particularly challenging for people possessing a limited number of frameworks.

*Wicked problems*, which Rittel and Webber (1973) identified as problems whose solutions change depending on how they are approached and by whom, are never fully resolved and do not involve binary answers. We suggest that *all* problems are wicked problems for the novice learner. Because the learner is not well-versed or rehearsed in the ways problems are identified and resolved, they may not know where to begin, or have the self-efficacy to start experimenting in an effort to refine their approach. Thus, we further propose that explicit teaching of and implicit exposure to different mental frameworks will equip learners with more, and eventually better, choices for problem resolution over time. Exposure to a greater variety of mental frameworks for problem solving also has the potential of increasing one’s confidence to begin problem solving tasks in the first place. The more rehearsal one has with frameworks, the more automated and rapid the ability to respond heuristically. We propose that the quantity of exposure should be matched by the quality and variety of approaches. We believe that the *Theory of Mental Frameworks* is unique in approaching problem-solving at all age levels and within all subject areas as a life-long learning process achieved through rehearsal and resulting in heuristics. The ability to effectively employ varying frameworks then, matters more than the specific subject matter or environment in which the problem may be tackled.

#### 1.1.1. The problems of problem-solving: tool access and use

To effectively solve any problem, whether simple or complex, it is essential to have the right tools, processes, and strategies in place. Although it is possible to solve problems without the proper tools, the process will likely be more challenging, less efficient, and the range of solutions more limited (Lowes, 2020). This paper identifies some of the mental frameworks that may be used to solve complex problems in particular. “Complex problem-solving is a collection of self-regulated psychological processes and activities necessary in dynamic environments to achieve ill-defined goals that cannot be reached by routine actions,” according to Dörner and Funke (2017, p. 6), which we label *mental frameworks*. Mental frameworks are processes that may be learned and accessed both implicitly (unconsciously) and explicitly (consciously), and which may be recalled automatically with practice. The researchers elaborate further on the broader problem-solving process, which, “combines cognitive, emotional, and motivational aspects, particularly in high-stakes situations” (p. 1,153). These emotional and motivational aspects require their own set of learned strategies, known as *coping* in more high-stakes situations, to support the explicit cognitive strategies involved. They are also supported through the cultivation of social emotional learning (SEL) skills that serve as “emotional rudders,” helping to guide problem-solvers through relational dynamics that accompany these processes, both in and out of school settings (Immordino-Yang and Damasio, 2007, p. 3). Importantly, complex problem-solving tends to involve some degree of collaboration or interaction and is not typically processed by the thinker in an entirely independent manner.

Everything learned can be defined as knowledge, skills and/or attitudes (Wiggins and McTighe, 2005) which De Houwer and colleagues explained are gained through experience and “ontogenic adaptation” due to “changes in behavior” based on “regularities in the environment” (De Houwer et al., 2013, p. 631). This means people’s belief systems about their world are modified by different kinds of experiences. By extension, problem-solving requires learning about, and then using, different mental frameworks that pull from those knowledge, skills, and attitudes learned. We propose that (a) for many learners much of this happens without awareness, and (b) one can become an expert problem solver with the intentional accumulation of frameworks gained through experience over time.

#### 1.1.2. The potentials of problem-solving: adaptive expertise

Effective problem-solving depends on the mental tools available, and the more tools that are utilized efficiently, the better the problem solving will be. While basic domain knowledge may be sufficient for simple or familiar problems, novel or complex problems require cognitive flexibility to consider relevant prior knowledge, cross silos of field understanding, and to generate new potential solutions (Miyake et al., 2000). Cognitive flexibility refers to the ability to mentally switch between different concepts in response to a specific situation or novel context (Scott, 1962) and relates to adaptive expertise (Carbonell et al., 2014).

The concept of “adaptive expertise” was first introduced by Hatano and Inagaki (1986) as a contrast to routine expertise. In this conceptualization, both adaptive and routine experts are successful in familiar situations, but when faced with novel situations, routine experts struggle, while adaptive experts demonstrate flexibility in thinking (Schwartz et al., 2005) and are able to apply their knowledge of what approaches to use, and when and how (Carbonell et al., 2014) to find an effective solution. Adaptive experts not only apply previously acquired knowledge and skills to new and unfamiliar situations, but they also modify or restructure that knowledge as needed to solve problems in new contexts (Hatano and Oura, 2003). This requires a deep understanding of a particular domain as well as the ability to transfer knowledge to new and diverse situations (Rittle-Johnson and Star, 2007).

Furthermore, effective employment of an appropriate adaptive strategy based on the specific problem at hand, requires an assessment of both cognitive and emotional conditions. “Indeed, emotion without cognition is blind and… cognition without emotion is vacuous” (Scheffler, 1977, p. 171). This suggests there is always an emotional element to any problem, and that our responses to them inherently involve emotional processing as well. This explains why some younger children have trouble with problem-solving skills as they may not have refined emotional intelligence or social regulation skills, which are highly related. Some mental frameworks are affectively oriented while others are cognitively oriented.

Research demonstrates that mental frameworks that employ the ability to pivot emotionally or involve attitudinal shifts, such as Dweck’s Theories of Intelligence (better known as Growth and fixed Mindset Theory; Dweck, 1999), Brackett’s RULER approach (Brackett, 2019; Brackett and Cipriano, 2020), or Costa and Kallick’s *Habits of Mind* Costa and Kallick (2000), compliment mental frameworks that focus primarily on cognitive elements, such as planning, assessing, or designing. Each contributes to the formation of a more robust toolbox, which enhances cognitive flexibility in problem-solving. Consequently, those with greater cognitive flexibility can become “adaptive experts” with the tools they possess as they respond to novel and complex situations, utilizing an effective balance of prior knowledge and generating new knowledge (Carbonell et al., 2014), which can become habituated over time. This ability to adapt and apply increasingly numerous and complex mental frameworks is growing in importance for both students and professionals as the need for critical and creative problem solving expands in the classroom and beyond (Carbonell et al., 2014).

By extension, the experience-expectant aspects of cultivating an “adaptive toolbox” for problem-solving focuses on actively developing additional thinking strategies and tools to aid in the process, according to Gigerenzer and Todd (1999). Furthermore, the researchers revealed that a key aspect when using an adaptive toolbox is the ability to recognize when a particular strategy is not working and switch to a different one, thereby using “fast and frugal” heuristics (Gigerenzer and Todd, 1999). Fast and frugal heuristics, therefore, enable individuals to make optimal decisions within the limitations of available time and knowledge. Accordingly, in order to effectively use an adaptive toolbox for problem solving, one needs to have sufficient familiarity with the tools and mental frameworks therein.

The strength of adaptive expertise, or adeptness of one’s adaptive toolbox, relies on both the quality and quantity of one’s exposure to a range of problem-solving frameworks. This includes both the conscious and unconscious use of tools, something which can be enhanced with intentional effort and conscientious instruction (Bayounes and Saâdi, 2022).

## 2. Theoretical framework

Piaget (1923) published, *The Language and Thought of the Child* in which he introduced the concept of mental schema. Mental schemata are mental structures upon which people build their knowledge of the world. Since its introduction, studies in fields as broad as philosophy (e.g., Nevid, 2007) and neuroscience (e.g., Ohki et al., 2023) have confirmed the existence of such thought organizing mechanisms in the brain (Ohki and Takei, 2018). This paper proposes to build off of Piaget’s ideas of mental schema, which are conceptual understandings, and will pull from available models of mental processing to consider a *Theory of Mental Frameworks*, a collective *grouping of schemata*. While schema generally surround semantic knowledge, such as the meaning of words, we use the term *mental frameworks* to imply an understanding of the ways people habituate thinking *processes*. Earlier work on problem-solving has made the distinction of problem-solving as a product versus a *process,* including Gross and McDonald’s work which stated that, “forward-looking science educators have decried the mistaken notion held by so many teachers in all curricular fields that problem-solving abilities are merely by-products of the memorization of the lesson or result almost automatically from learning a set of facts,” Gross and McDonald (1958, p. 259). Just as people use the heuristics of schematic knowledge to forge implicit shortcuts in thinking and decision making (Vazsonyi, 1990), we propose they also use mental frameworks when they approach problem-solving through explicit processes.

Jean Piaget’s theory of mental schema suggests that people can “expand” their mental schema by layering multiple levels of understanding to different concepts. If a person had only known one kind of “dog” in their life, the mental schema of a dog was limited. Others who had known many kinds of dogs had many ways of understanding dogs, displaying a broader mental schema, including other breeds, stuffed animals, cartoons, and other renderings. Mental schema moved beyond being a psychological theory of learning to a neurophysiologically demonstrable construct, established firmly in the literature. Relatedly, one of the most vibrant areas of neuroscientific research for the past two decades relates to the study of the neural correlates of consciousness (Chalmers, 2000). People now take mental schema for granted and it is perfectly normal to presume that others have different relationships with concepts (schema) based on their prior knowledge of the world.

Research on Theory of Mind, or the way people read each other’s minds based on perspective-taking and prior knowledge, furthered the belief that mental schema could vary between individuals (Baron-Cohen et al., 1985; Baron-Cohen, 1991). The understanding that mental schema are not always shared was supported by work in psychology in the 1980s and 1990s, using false-belief task experiments (e.g., Wimmer and Perner, 1983). An understanding of Theory of Mind revealed that different people’s life experiences change the knowledge base they have for meeting challenges in the world. These differences are based on unique experiences and habituated responses developed over time, namely heuristics and biases (conscious and unconscious), and behaviors. If we accept that people’s mental schema are different, then it is likely their mental *frameworks* for problem solving may also differ (Chen, 1999).

Models of thinking exist across all academic fields and are used to identify the ways people contemplate, negotiate, and manage their worlds (see *Mental Models: Learn How to Think Better and Gain a Mental Edge* by James Clear, 2023 as an example). Mental frameworks to approach problem solving in education have existed for decades and are most recognizable as “problem-based learning” strategies [see Cindy Hmelo-Silver’s many publications as examples, (Hmelo and Cote, 1996; Hmelo-Silver, 2004; Hmelo-Silver and Barrows, 2006)]. While we agree that “all models are wrong, but some are useful,” according to Box's (1979) famous aphorism, we think that the transdisciplinary selection of mental frameworks from mind (psychology), brain (neuroscience), and education (pedagogy), often referred to as MBE, can lead to a powerful toolbox of options for all problem-solvers, be they teachers or learners.

To explore this further, we rely on the premise that how and to what extent models and frameworks are easy to employ, often determines their utility. We offer that frameworks which are easily accessible, highly transferable, and generalizable, represent good models that will be used often. The following three sections of the paper explain the nature of problem identification and solution seeking to gain an appreciation of the multi-faceted ways problem-solving can be taught in schools. This is followed by examples of mental frameworks from MBE, and a discussion of the possible utility of this Theory in educational practice.

## 3. Problem-solving and the brain

Learning to think in order to resolve problems involves multiple cognitive processes and uses a complex combination of neural networks in the brain, which vary depending on the task at hand (Alchihabi et al., 2021). For example, while the recollection of the meaning of a word is a relatively simple cognitive process on the surface, it is actually highly complex (Dreyer and Pulvermüller, 2018). Recalling the word meaning is one thing, however actually using it will also depend on an understanding of the contexts in which it may be employed (Ferreira et al., 2015), how it combines with other words (Grisoni et al., 2017), and who is present in the use-context (Renoult et al., 2019), among numerous other dynamic factors. Calling upon mental frameworks follows a similar process but is far more complex than recalling words. This invites exploration into how unconscious heuristics may be altered by a learner’s conscious decision to learn about different mental frameworks.

Benson (2016) suggested that most cognition, including problem-solving, is framed by bias, which in turn is driven by unconscious heuristics. This type of thinking involves dozens of neural correlates and thousands of synaptic processes spread out over different cortical areas (Gnedykh et al., 2022). To find the meaning of a word in semantic memory conjures dozens of simultaneous autobiographical memories about its existence in one’s life (Teghil et al., 2022; Mace and Kruchten, 2023), networks to articulate this to another person using words (Wank et al., 2020), and possibly even more, if written (James et al., 2015). Semantic memory is only one of dozens of complex processes involved in the brain’s understanding and resolution of problems.

Problem-solving in the brain involves general cognition (memory, attention, executive functions; Miguel et al., 2023) as well as domain specific knowledge, such as recall of mathematical formulas, art genres, or periods of history. Sub-areas of memory include working memory (Emch et al., 2019); both procedural (van den Berg et al., 2023) and declarative (Sarathy, 2018), long-term memory, and sub-systems other than autobiographical and episodic memories, such as emotional memory tracts (Engen and Anderson, 2018). Processes related to attention include alerting attention systems (Zabelina et al., 2019), orienting attention systems (Spadone et al., 2021), and sustained attention (Fisher, 2019). Executive functions measure a range of abilities (Burgoyne and Engle, 2020); inhibitory control (Bajo et al., 2021); and cognitive flexibility (Balázs, 2019). Domain specific processes are also needed, which has to do with specialized field knowledge (e.g., Neubert et al., 2017).

As there is no cognition without emotion, there is also an abundance of literature which considers the role of affect on problem-solving. Pekrun and Loderer have led work on emotions and learning in academic settings for decades Pekrun and Loderer (2020), and their most recent review of the emotions that are influential in learning show important links between “multiple representations and perspectives” (p. 373). Furthermore, a large amount of literature explains how stress influences learning in negative as well as positive ways, related to both individual and group learning (e.g., Avry et al., 2020).

People approach problems in a number of ways, including through the use of analogical thinking to understand a current situation (Parsons and Davies, 2022), and procedural strategies to resolve problems (Sokolowski et al., 2023). Other research looks at how a person makes inferences while speaking (Jara-Ettinger and Rubio-Fernandez, 2021) to fill in gaps in knowledge, or uses social cues to learn the intentions of another (Henry et al., 2021). Some research studies the brain as it experiences “insight” versus ordinary problem solving, by gauging whether people approach problems using simple visual networks compared with higher order thinking networks (Lin et al., 2021). Yet others like Shpurov et al. (2020) and Prince and Brown (2022), seek to understand what changes in the brain when an individual approaches a problem on their own versus within a group setting. Hong and Page found that collaborative work among people who approached problems differently was actually superior to that of individual expert problem solvers Hong and Page (2004). These many different sub-elements in the neural correlates of problem-solving suggest that different combinations of thinking tasks are used during different approaches to problems.

### 3.1. Prioritizing problem-solving *processes* over *products*

If the collective goal of schooling is to prepare students for the future by encouraging them to think for themselves, master skills and knowledge, and innovate, as governments and organizations ranging from the U.S. Department of Education to the Organization for Economic Co-operation and Development (OECD, 2019) suggest, then learning to resolve problems using critical and creative thinking is to be expected as a regular part of teaching. Evidence shows that when the goal of problem-solving achieved through critical thinking is met, student outcomes are better, the transition to adulthood happens more seamlessly, and success in its various forms unfolds for more types of students and in more contexts (e.g., Frey et al., 2005; Linares et al., 2005; National Commission on Social, Emotional, and Academic Development, 2019; OECD, 2019). However, these goals and their associated positive outcomes tend to be at odds with many existing educational practices and systems, and may be harder to implement (Ahmadi et al., 2019). Decades of curricular focus on critical thinking and problem-solving have revealed challenges that span developmental ages and stages and various types of educational approaches (e.g., Ahmadi et al., 2019), which often do not explicitly teach mental frameworks for problem solving, but rather implicitly attempt to embed them in classroom activities.

Thinking “outside-of-the-box” which is associated with critical thinking and problem-solving, is often non-linear, as seen in most of the mental frameworks shared in this paper, such as Design Thinking, which has a more circular and iterative process (Serrat, 2017), or holonic thinking which requires constant changes in perspective taking (Tokuhama-Espinosa, in review) that underpins cognitive flexibility. This is in contrast to the single correct answer possibilities expected on a typical standardized test (Au, 2011). Divergent and creative thinking takes time and patience to generate, and depending on the subject matter, it does not always yield a singular answer or solution. Thinking outside the box centers the learning on the learner, and heavily depends on things like context (e.g., Amabile, 2018), past experiences (Acar et al., 2019), the relationship between student and teacher (Hattie, 2012; Martinez et al., 2016; Wentzel, 2016), one’s social and emotional skills (Immordino-Yang and Damasio, 2007; Jones and Bouffard, 2012), and the specific risk and protective factors of a given student (e.g., Ellis et al., 2017).

These studies suggest that problem-solving cannot be nurtured in a one-size-fits-all process (e.g., Davis and Autin, 2020), but rather varies by individual and context. As a result, while schools do teach problem-solving, the realities of doing so for different types of learners across subjects, in age-appropriate ways, against the backdrop of state and national accountability measures, and within the constraints imposed by the design of a typical school day (e.g., class length, variability in student/teacher ratios) means that problem solving in schools is often reduced to single solution activities (Au, 2011) and/or infrequent activities that are not presented with enough regularity to induce the “fast and frugal” heuristics mentioned earlier. Despite a growing awareness of the importance in learning of mental processes and individual contextual factors of each student, current problem-solving in schools is still largely focused on getting to a specific, single final product (e.g., correct answers on a multiple-choice test). This problem offers an opportunity to model what society collectively hopes to teach—problem-solving—in service to the wellbeing of children and with the ultimate goal of improving teaching and learning.

### 3.2. Identifying and resolving problems

Many readers are familiar with the questioning stage of young children, which can start as early as two or three-years of age, in which children respond to every answer by asking “Why?” All children around the world go through this stage (Mackey, 2023) suggesting that questioning is an innate quality shared by people of all ages (Seyferth et al., 2022). Research suggests it is much harder to come up with a question than to answer one (Marzano et al., 2001), and many teachers intuit that there is a higher level of thinking involved in question formation than in question answering. The more complex nature of problem identification is also born out in neuroscientific studies. Just as multiple-choice questions are easier to answer than open-ended questions, involving fewer complex networks in the brain (Zhang et al., 2021), the retrieval of information to answer a question is less neurologically complex than formulating one (Stoewer et al., 2022). As a teaching strategy, Rothstein and Santana encourage us to *Make Just One Change: Teach Students to Ask Their Own Questions* Rothstein and Santana (2011). This is similar to real world problem-solving in which awareness of one’s condition is the first step toward bettering that condition (Asy'ari and Ikhsan, 2019). Prior to resolving a problem, one must know the problem exists in the first place. Melles et al. (2015) call this “problem identification” and consider it the first step in design thinking which moves toward authentic solutions to real world problems.

Some people can tackle problems easily, even with little or no prerequisite knowledge (Salmon-Mordekovich and Leikin, 2022). That is, faced with any problem, they know steps to begin the resolution of the problem or to find creative responses. Others struggle to approach problem-solving, even within their field of expertise. They may have a hard time because they do not see the problem to be solved (Dandan et al., 2022). This suggests that identifying what constitutes a problem and knowing where to begin are difficult, often complex, and involve higher order thinking.

Both finding and resolving problems are teachable skills, and for decades teachers have been tasked with the responsibility for developing problem-solving skills (Weir, 1974; Dilekli and Tezci, 2022). Many excellent teachers manage to introduce one or more mental frameworks to facilitate problem identification and resolution in class activities, and methods such as inquiry-based learning for problem solving have shown superior learning outcomes (Hala and Xhomara, 2022). As different types of problems require different approaches, we suggest that the introduction of multiple mental frameworks in each class situation would benefit long term thinking skills in students.

Several of the mental frameworks ripe for inclusion in this review come from the learning science fields of mind (psychology), body and brain (genes and neuroscience), health (mental and physical wellbeing), and education. To illustrate the ways different mental frameworks from distinct fields may contribute to a student’s toolbox of mental framework options, we will first discuss four popular frameworks for problem-solving found in education, then describe three models from psychology, followed by four frameworks from neuroscience.

## 4. Existing mental frameworks from education

Mental frameworks in education span a broad range of contexts. Some important models not discussed in this paper in detail, but worthy of consideration, include the sophisticated classification structures related to mental frameworks for thinking, such as Project Zero’s visible thinking routine toolbox (Ritchhart et al., 2011), and for social–emotional learning, such as the Collaborative for Academic, Social, and Emotional Learning (CASEL, 2020) “wheel” framework. Other important mental frameworks often used by teachers include the Theory of Multiple Intelligences (Gardner, 1983) and the Quadruple Helix model to motivate global citizenship (Socher et al., 2021).

Educators have looked to other spaces such as the worlds of business and design in an attempt to solve the problem of how to teach problem-solving in schools (e.g., Noweski et al., 2012). Various models have been adopted and applied (e.g., Davis and Autin, 2020; Foster, 2021; Kijima et al., 2021) to include *Design Thinking* and *Understanding By Design*. Like other problem-solving models, however, these frameworks rely on important professional development and educator acceptance (Schell, 2014). Mental frameworks borrowed from business require contextual adaptation in order to be generative in school contexts.

Four mental frameworks from education that are supported by dozens, if not hundreds of studies, include those that seek **shifts in attitude** to improve the likelihood of learning, both in formal and informal contexts (e.g., ***Habits of Mind***); leverage good **planning** to resolve problems (e.g., ***Understanding by Desig*n**); employ empathy and cognition through **design thinking**; and take stock of problem elements through **assessment** (***Compass*** and **SWOT** activities). All may be applied starting in early childhood and can be developed over the lifespan.

### 4.1. Shifts in attitude for problem-solving: *habits of mind*

Developed by Costa and Kallick over the past 40 years, *Habits of Mind* Costa and Kallick (2009) is a list of 16 ways to improve the likelihood of life and school success, and has been used by U.S. school districts since 1998. According to the authors, “a ‘habit of mind’ means having a disposition toward behaving intelligently when confronted with problems,” (Costa, 2010, p. 1). This design pre-dates many of today’s accepted ideas about motivation, learning, and problem-solving, which are all part of the habits.

Art Costa’s work on intelligent behaviors Costa (1981) lead to a 1991 publication bringing together researchers, philosophers, and cognitive psychologists to find consensus on how to develop thinking (Costa, 1991). The Habits of Mind were formed in collaboration with Bea Kallick’s contributions, which showed that each of the 16 habits could be designed and assessed as learning experiences (Costa and Kallick, 1995).

Habits of mind:

- Persisting (not giving up in the face of difficulty)
- Managing impulsivity (self-regulation)
- Listening with understanding and empathy (the ability to take others’ perspectives)
- Thinking flexibly (not having a fixed mindset)
- Thinking about your thinking (metacognition)
- Striving for accuracy and precision (not settling for “good enough” but rather striving for the best)
- Questioning and problem posing (identifying areas in need of improved or better information)
- Applying past knowledge to new situations (learning from the past)
- Thinking and communicating with clarity and precision (generating and sharing ideas with accuracy)
- Gathering data through all senses (using sight, smell, taste, touch, and hearing to learn about the surroundings)
- Creating, imagining and innovating (always seeking ways to make things better)
- Responding with wonderment and awe (finding joy in everything)
- Taking responsible risks (not being compliant)
- Finding humor (not taking oneself or the world too seriously)
- Thinking interdependently (1 + 1 = 3; using the wisdom of the group; know yourself by knowing the other)
- Remaining open to continuous learning (openness)

There is evidence that people who adopt the 16 habits are better at approaching problems because they are open, do not give up, seek alternative pathways to answer questions, and use all tools available (Alhamlan et al., 2018). Research reveals benefits when the habits are adopted collectively as well as when they are used individually (Costa and Kallick, 2009). Additionally, upon review we found they overlap with elements of other focuses of learning, including executive functions (Saleh Al Rasheed and Hanafy, 2023), social–emotional learning (Alexander and Vermette, 2019), the Big Five Personality trait of openness (Abdellatif and Zaki, 2021), and the Mind, Brain, and Education Principles and Tenets (Tokuhama-Espinosa, 2014). The 16 habits of mind are useful in addressing problems that require a shift toward positive thinking (Anderson, 2021), and for problems in which the learner likely knows the answer but does not have instincts about where to begin (Jones, 2014). Costa and Kallick suggest that the habits of mind should be taught from early childhood, but can be learned later in life as well, and are constantly refined throughout the lifespan. This attitudinal approach to problem-solving is appropriate for all subject areas and for life in general.

### 4.2. Planning for problem-solving: *understanding by design*

*Understanding By Design* (UbD; Wiggins and McTighe, 2005) is a mental framework in which teachers and learners always begin with three simple questions: (1) What is the objective? (2) How will I evaluate? (3) What do I do? In this model, the learning begins with the end in mind: *Where do I want to be when this process is finished? How will I know I have been successful in meeting my goals?*

The first step in UbD is to identify the objective. It is a useful mental framework when clarity is needed to shed light on a problem, and to establish the “why?” behind instruction (e.g., *why learn this?, why do the assignment?; why is this important?; why meet over this topic?*), which is also known as “root cause analysis” in psychology (Okes, 2019). In a classroom setting this tends to support collaboration between teachers and students toward a co-constructed curriculum, as the overall objective of assignments is made explicit. The second step identifies the many ways people can evaluate advancement toward the objective, ensures everyone shares the same understanding of success criteria, and commits the group to one clear and transparent tool. For example, a school may have a goal of “academic excellence” but some may think excellence means high test scores, others may think it is school harmony, and yet others might think it means having a well-rounded or happy student body. Shared criteria increases the likelihood of achieving objectives (Moss, 2022). Once the objective and the evaluation tools are agreed on, the final step is to plan the activities and identify needed resources. This is a primary sticking point in education, as many schools plan activities before defining objectives and plans for assessment. When this happens, people often end up evaluating the activities rather than any progress toward the shared objective. Additional problems arise when there is a mismatch between the objective and the evaluation tools and/or activities designed, and this can impact acceptance and motivation.

UbD is a useful mental framework for problem-solving at all levels of education and can, in fact, serve as the default starting point when approaching *any* problem in need of clarification, independent of whether the actor is the student, parent, or teacher. If one begins by asking *why the problem should be solved* in the first place, this process of objective identification sets the solver in motion to follow a framework that supports effective problem-solving. Moreover, by beginning with UbD, people clarify their own biases and presumptions about the benefits of a given approach before searching for solutions. Teachers can model this approach for students as early as preschool and do so by simply incorporating a short conversation about why each new learning objective is important, how it will be measured, and how it will be learned.

### 4.3. Problem-solving through *design thinking*

A third framework worthy of consideration is *design thinking*. In Brenner and Uebernickle’s model, Brenner and Uebernickel (2016) of design thinking there is a seven-step process. These steps begin by establishing empathy for the people who will be most affected by the new design, usually the end-user. Once empathy is established, one can then (a) define the problem, (b) determine the root causes of the problem, (c) brainstorm or develop alternative solutions, (d) select the best solution, (e) implement the solution, (f) evaluate the outcome, and (g) reassess the problem. The end result of a positive design thinking experience, according to Panke’s summary Panke (2019), is to increase collaborative decision making, promote playful learning, reduce cognitive bias, create conditions for flow, foster meta-disciplinary collaboration, nurture creative confidence, induce productive failure, and increase resilience.

Design thinking has roots in engineering and business (Von Thienen et al., 2018), and combines problem-based learning with inquiry and project-based learning, to devise authentic learning experiences. Design thinking is particularly beneficial when the goal is to solve certain types of wicked problems in groups, like those associated with creating or fixing a physical object, policy, or program. While typically associated with older students, design thinking may be explicitly and implicitly used in early-years-education and might require nothing more than the props of the environment. For example, a preschool teacher can point out the difficulties of a person trying to mount the stroller over the sidewalk when there is no ramp (“Poor lady! How hard it is to get a stroller up and over that curbside!”) and ask the kids what could be done about it. Panke (2019) notes that design thinking is both a process as well as a mindset, however. While great for problem solving, design thinking can also be a source of frustration or anxiety for participants unfamiliar with collaborative problem solving under design thinking conditions, which is why exposure to design thinking early in life may help reduce resistance later.

### 4.4. Assessment of problem situations: pairing *compass and SWOT*

The Compass activity fits within Harvard’s Project Zero’s “Thinking Routines” (Ritchhart and Church, 2020) and is a successful framework for assessing personal perspective on problems. The SWOT analysis method is thought to have been born in business education programs in the 1960s (Kaplan and Norton, 2008), and was embraced by industry in the 1970s (Andrews, 1971). It has been used as a problem-solving tool in education since the 2000s (AlMarwani, 2020).

Designed to facilitate self-assessment and the assessment of situations, the Compass Activity is a mental framework which asks the learner to think of the North, South, East and West as follows:

- **N**orth stands for **Needs**
- **S**outh represents **Steps** to take
- **E**ast means **Excitement**
- **W**est demonstrates the problem-solver’s **Worries**

Before beginning to resolve the problem, the problem solver considers their own emotions around the steps to problem-solving, by assigning answers to what they need, what steps they should take, what they are excited about, and the worries they have. Children as young as three can be coached into self-assessment in this way, and the tool remains exactly the same for adults facing problems. Once the problem solver has decided on a resolution, they can then conduct a SWOT analysis. A SWOT analysis asks the problem solver to consider their situation based on the chosen resolution:

- What **Strengths** does this resolution provide?
- What **Weaknesses** have been created or remain?
- What **Opportunities** does this offer moving forward?
- What **Threats** can hinder true problem resolution?

Engaging in both the Compass activity and the SWOT analysis encourages the problem-solver to be both introspective, and to “zoom out” for perspective, which changes the nature of the problem (Minsky and Aron, 2021). For example, if one needs the teacher to give more help (external), the problem is different from one needing more time to do the work (internal). Similarly, if a solution exposes a weakness in leadership (external), that is different from thinking one’s computing skills are weak (internal). The locus of control in approaching the problem changes, based on this internal and external assessment. Learners who habituate the Compass Activity and SWOT learn that no resolution is without its conflicts, and that those can have roots in *who* is presumed to have control over the problem resolution.

This broader mental framework helps learners identify and embrace what they are working toward and excited by, while also pinpointing the worries and threats that may accompany a successful project. Ostensibly, this framework encourages a thinker to consider their emotions while also identifying actionable steps to take, thereby explicitly connecting emotion and cognition in decision making. This model is particularly good for problems in which the learner has low motivation and needs to be reminded of the benefits of resolving the problem, as it encourages a focus on strengths and opportunities. It may be used by teachers and learners to distill a bigger problem into manageable “bite-sized” pieces in any subject area (AlMarwani, 2020).

## 5. Existing mental frameworks from psychology

Some mental framework examples from the field of psychology that show excellent results, but will not be discussed here include *Solution Stories* (Kelley, 2018), which are based off of Vygotsky’s Social Constructivism Vygotsky (1978), Pekrun’s Control Value Theory of Emotions Pekrun (2006), and Lerner’s work on human development Lerner (2006); the *Monsen Problem—Solving Model* (Monsen and Frederickson, 2016); and *Cognitive Decoupling* (Koichu and Leron, 2015), which is based on hypothetical thinking, mental representations, and working memory capacity.

Three examples from psychology will be presented. We will first examine the ***Cognitive Bias Codex*** and show how it is used to problem-solve, based on types of information intake. We will then discuss how problem-solving functions as an ongoing negotiation between **challenge and threat**, and how one’s self-perception as a learner influences successful problem-solving through **growth mindset** maintenance.

### 5.1. Constraints on perception and decision-making during problem solving: *cognitive bias codex*

Benson’s Cognitive Bias Codex (“CBC”; Benson, 2016) represents an interesting mental framework to aid in teaching and learning and serves as a psychologically grounded bridge from the educational models mentioned above to newer frameworks from neuroscience, which follow. Chronological in its structure, the Cognitive Bias Codex is one way to explain that information, meaning, and time create constraints within which our brain understands the world and thereby develops heuristics and biases. Benson suggests signals are detected in the environment, whereby personally relevant meaning is assigned to them based on an individual’s prior experience. Next, a decision is made, often automatically or without conscious awareness, based on that primarily subjective meaning. The result of that decision is then experienced. The memories created through this process are fed back into the system to influence subsequent iterations of this process.

While by no means the only taxonomy of bias (also see Tversky and Kahneman, 1982; Oreg and Bayazit, 2009; Gigerenzer and Gaissmaier, 2011; Korteling et al., 2018) the CBC suggests that our interaction with the world is always influenced by what we already know. What we already know (or do not yet know) may hinder problem-solving. For example, there may be too much new information with which the learner is unfamiliar. Other times problem-solving is hindered because a learner lacks meaning or context for the learning. In a third case, problem-solving may be hindered because a student has too little time to respond thoroughly. Finally, Benson suggests that in other instances, one’s problem-solving skills (or lack thereof) are due to an inability to know what is important or to prioritize information. This framework suggests people are often unaware of the biases under which they perform daily routines, including problem solving, because they are driven by observable, however unconscious, heuristics grounded in prior experience.

The CBC is helpful in problem-solving when it is unclear why progress is not being made, and it can also aid a learner in identifying biases of which they were previously unaware. In a hypothetical example, let us presume there is a woman who is in charge of environmental issues at her company. She is asked by her boss to recommend priorities for the coming year.

“Everything,” she answers.

“Yes, everything is important,” responds the boss, “but what should we prioritize?”

“Everything,” she says again.

“But what, specifically, would you recommend we give most of our budget and attention to?”

“Everything. The environment is everything, so everything is important.”

“That’s precisely why I would like your opinion. We can’t do everything, so I’d like you to suggest what is most important.”

“It’s all important.”

“Yes, it is all important. But where should we focus? The oceans? Plastics? Toxins? Carbon emissions?

“Yes.”

“Which?”

“All of them are important.”

Despite being the expert on the environment in the office, with awareness of many environmental challenges, the woman is unable to prioritize them. What keeps intelligent people from being able to resolve the problem at hand (e.g., plan the budget and agenda for the coming year)? There are four primary answers, according to Benson. Sometimes the ability to resolve a problem is due to “analysis paralysis” in which too much information is presented to be processed all at once. On the CBC, this is seen as a “Too Much Information” problem (A). It is also possible the woman was unclear about what her boss needed from her. *Was this a report? A list? A budget?* She might not have had enough meaning (B) to respond. In other cases, some, but not all people with a high level of *content knowledge* and sufficient *communication skills* are familiar enough with the problems of their field that they can consider patterns of past responses and use them to approach new problems but cannot do this quickly. This may result in difficulty responding in the “Need to Act Fast” quadrant. The speed of reply is related to the familiarity of responses from the past (C). Finally, in other cases the woman might have had access to all the right information, and understood it, but was unable to prioritize it (D).

The Cognitive Bias Codex may be new to teachers but it relates to situations visible in all classrooms and all age levels. Students may be unable to resolve problems because they have too much information and do not know how to order the information (A). They may take in the information but have insufficient prior knowledge upon which to scaffold new understanding (B). Perhaps the most common problem, also identified by Benjamin Bloom in 1968, is that there is not enough time for smart students to make their way through the information, resulting in hurried answers which are insufficient (C). Finally, many students learn vast amounts of content shared in the classroom and hold it long enough to pass tests, but do not retain it all (D) for reasons ranging from a lack of authentic context, strong mental schema, or association to other prior knowledge. The CBS is a useful framework for problem-solving at all age levels, within all topics, and useful beyond the school years.

### 5.2. Reframing and problem solving: *challenge and threat*

The “Threat versus Challenge” outlook is a problem-solving framework for appraising life’s circumstances to the benefit of performance and outcomes (Lazarus and Folkman, 1984). Learning requires a great deal of energy. Approaching problems as challenges rather than threats results in physical bodily changes, permitting the problem solver to be more efficient with their limited energy. If a student believes in their own ability to tackle a problem, however challenging, they experience fewer negative physical, emotional, and psychological outcomes (e.g., Mitchell et al., 2019; Wormwood et al., 2019). This does not mean that they are fully equipped to solve a given problem, but it does mean that with mind, brain, and body in greater balance they have more energy to recruit and access the needed resources (e.g., knowledge, skills, social support, instrumental supports) and manage stress which can otherwise interfere with thinking. By approaching problems as challenges and not as threats, the equilibrium of the student becomes a protective factor for successful, open-minded problem-solving.

Originally based on coaching models, threat versus challenge has been widely studied in athletic settings (e.g., Mitchell et al., 2019; Meijen et al., 2020). Engaging in this mental framework activates the parasympathetic nervous system, which in turn, allows the executive networks of the brain to preside over the sympathetic nervous system, thereby down-regulating and calming the limbic system (e.g., Sicorello et al., 2021). Consequently, if a student is afraid of something (danger/threat), they are more likely to retreat, but if they view it as a challenge (opportunity), they are more likely to spring into action, and seeing something as a surmountable challenge increases the likelihood of problem resolution (e.g., Lazarus and Folkman, 1984; Tomaka et al., 1993; Mitchell et al., 2019). This perspective makes room for divergent and creative thinking because the brain tends to treat challenges with approach-style responses (e.g., *What action can I take?, What do I know about this?, What help might I recruit?*), and threats with retreat or survival-oriented responses (e.g., fight/flight/freeze).

Using the challenge versus threat mental framework to resolve problems is particularly useful when approaching new or unfamiliar problems (Espedido and Searle, 2020). This approach is also supportive when the problem-solver has a previous self-perception of being “bad” at the type of problem being resolved (Buffone, 2015). This mental framework of applying a cognitive reappraisal to a problem before approaching it can have a direct impact on one’s problem solving abilities (Eastcare and Greenville, 2019). Costa and Kallick suggest that approaching the world and its problems with “wonder and awe” Costa and Kallick (2009) can habituate a challenge mentality, reduce threat perception, and is a skill that can be taught to very young children, but should be rehearsed across the lifespan in as many contexts as possible.

### 5.3. Growth mindsets and problem-solving: *Dweck’s mindsets*

A mental framework similar in premise to “challenge and threat” is that of Dweck’s mindset theory (Dweck, 1999, 2006), wherein a growth mindset—one’s positive belief about their own ability to grow and improve through incremental effortful action—influences [academic] outcomes. By contrast, in Dweck’s model, a fixed mindset-oriented person believes that they were born being good or bad at certain elements of learning (or particular subjects) and does not see value in expending incremental effort designed to help them improve bit-by-bit (Brougham and Kashubeck-West, 2018). There is also extensive research showing that mindsets are malleable, and that a growth mindset can be improved and developed with intervention (Haimovitz and Dweck, 2017; Seaton, 2018; Zeeb et al., 2020). This explains why the internal mantra when facing a hard problem of “I cannot do it *yet*” is of such importance in growth mindset cultivation.

There is value in growth mindset training for educators and learners (Blackwell et al., 2007; Brougham and Kashubeck-West, 2018; Sarrasin et al., 2018) which relates directly to problem solving. Research on mindsets in educational settings has demonstrated that the mindset of the teacher can be as impactful (if not more so) as that of the student, in terms of a student’s beliefs about their own abilities in the classroom, and ostensibly, to solve-problems (Seaton, 2018; Canning et al., 2019; Frondozo et al., 2020; Richardson et al., 2020). First, a person with a growth mindset tends to view problems as opportunities, which permits them to face challenges incrementally, rather than succumbing to a counterproductive fear of failure or overwhelm. Second, problem-solving quality is enhanced because a growth mindset offloads demands on neurological, psychological, and physiological networks permitting critical thinking to occur (Ng, 2017: Sarrasin et al., 2018). These first two points result in a shift—rather than feeling defeated and depressed by a problem, people with growth mindsets consider them as opportunities to grow.

Using the mental framework of a growth mindset for problem-solving is best used when a positive reappraisal might be helpful, or an emotional or cognitive block is getting in the way of progress. Growth mindsets can be cultivated with the youngest of children and developed throughout the lifespan. It can help a student find motivation and serves to enhance physical and mental wellbeing in learning and is often a key element in the development of resilience.

The *Cognitive Bias Codex*, *Challenge and Threat*, and Dweck’s *Mindsets* serve to link the educational mental frameworks of the *16 Habits of Mind*, *Understanding By Design*, *Design Thinking*; and *Compass and SWOT* to the newer mental frameworks developed in just the past decade that come from neuroscience, which we turn to next.

## 6. New frameworks from mind, brain, and education

In addition to problem-solving frameworks drawn from education and psychology and used in the classroom, other learning sciences, such as neuroscience, may also offer important models. In this section we will consider the value of approaching problems from a **transdisciplinary perspective**; using **holonic thinking** to contextualize conceptual learning; employing knowledge of how the brain organizes information into the **five pillars** of symbols, patterns, order, categories and relationships; and leveraging **meaning making** strategies to make sense of context and bring authenticity to problem-solving. Each of these new mental frameworks from Mind, Brain, and Education is explained below briefly.

### 6.1. Perspective taking in problem solving through *transdisciplinary thinking*

Transdisciplinary thinking is an approach to problem solving that values the use of information from multiple fields. The belief is that the more, good information one has to resolve a problem, the better (Tokuhama-Espinosa, 2019). Studying domain problems like how to teach math or language, and other difficult problems in education, like student motivation, how to differentiate students based on their needs (and strengths), or ways to get children to be stewards of the environment, all require transdisciplinary thinking. It is now clear that there are few problems in the world that are better resolved using a single lens, framed only by one field of study, rather than by using multiple lenses, incorporating perspectives from various fields that “embrace the ‘mess’ of diversity,” (Kenter et al., 2019, p. 1,439).

Transdisciplinary studies were promoted by the Romans and were popular throughout the Middle Ages reaching a height with DaVinci’s Universal Man in the 1500s, which signaled the intellectual peak of integration of distinct fields like the arts and sciences. Beginning with the Industrialized Age in the late 1770s, jobs became more and more siloed and specialized (Nicholls and Murdock, 2012). Hyper-specialization was celebrated more than universal, transdisciplinary thinking throughout the 1940s and 1950s. However, the 1960s brought pushback against siloed ways of thinking, and it once again became popular to think about problem-solving using multiple lenses, with a renewed interest in transdisciplinary thinking at the forefront of debate (Jantsch, 1972).

Transdisciplinary thinking reminds problem-solvers to continually seek different perspectives and recognizes that different fields employ varying tools to measure and resolve problems (Yeh, 2019). It encourages a de-siloed approach to thinking that supports the problem-solver through a process of challenging their assumptions and considering a range of explanations, and honors the interconnected nature of everything (e.g., cognitions and emotions; genes and environments; risk and protective factors; individuals and groups, etc.). In facing the many kinds of problems that exist in the world (e.g., climate change, pandemics, poverty, war) and in the classroom (e.g., student motivation, community wellbeing, social engagement), transdisciplinary thinking asks problem-solvers to take the perspective of different field professionals to find answers. *How might an economist respond to a problem, as opposed to an environmentalist? How would a teacher respond as compared to a parent? A novice teacher compared to a master-educator? A social scientist* versus *a physicist?* By taking on different field perspectives in this way, problem-solvers are likely to identify solutions that would not be visible from a single disciplinary view (Swayne, 2020). Transdisciplinary thinking may be used in any realm to solve all types of problems and is particularly helpful in solving wicked problems and problems with several conflicting solutions.

Transdisciplinarity is challenged by the current design of education in which school is divided into subject areas. As a mental framework accessible from the earliest stages of development, it can, however, be developed by even very young children using a perspective taking approach to learning (e.g., Hodges et al., 2018) in which students are asked to play the role of different actors in problem-solving. Children can role-play various perspectives from around the age of three or four (*How would the shop keeper respond here? What would the mayor say? How would the children react?*). This can evolve from actor to disciplinary thinking (*What does biology say about this? What does philosophy propose? How would an environmentalist react?*) over time.

### 6.2. Problem-solving by examining all parts, macro to micro: *Holonic thinking*

An extension of transdisciplinary thinking is *holonic thinking* in problem-solving, which means appreciating that everything in the natural world can be considered a part as well as a whole (Esposito, 1976). The idea was derived from the Greek “holos” meaning whole, with the suffix “on” which, as in proton or neutron, suggests a particle or part (Edwards, 2003). A child is a whole unto themself, but she is also a part of a family, a school, a soccer team, and a classroom. A home is a single entity, but it is also part of a neighborhood, community, or town. Your brain is a whole, but it is also a part of your body. Holons can always be considered as smaller parts, or larger “wholes.”

Edwards (2003) suggests that the idea of holons has been around since the Middle Ages and was used to explain the spiritual connection between all living things. *Holonic thinking* was most famously referred to in *The Ghost in the Machine* (Koestler, 1967), and in the 1990s, was introduced in engineering to make solutions to problems more agile, by changing the way each piece fit into the larger whole (Van Leeuwen and Norrie, 1997). Most recently holonic thinking has been used to explain educational practice by Tokuhama-Espinosa and colleagues, when describing how children learn to write (in review). Breaking down the complexity of writing into its smallest parts (letters, phonemes, and so on), then bringing each lesson back to a more macro level (i.e., by showing how vocabulary building, spelling, grammar lessons and other aspects of writing come together to create the whole of writing), make the process (problem) of learning to write more manageable–holonic thinking in action.

Holonic thinking might be applied to the problem of teacher education and continued professional development (Tokuhama-Espinosa and Borja, 2023). There are numerous elements to teacher education, each a world unto itself. Some focus on planning, others on evaluation, and yet others on activities, or technology, or information about how the brain learns. All of these aspects are important. Each feature of teacher education can be broken down into smaller parts as well as viewed as a part of the larger whole, and considered through the lens of what a given teacher specifically needs. It may be broken into categories of skills, knowledge, and/or attitudes or learning formats (e.g., online, in-person, ongoing versus workshop-based), and so on.

Holonic thinking can be used as a mental framework for problem-solving as holons change the perspective on the object(s) within the problems, placing a spotlight also on the *relationships* between aspects of a holon and its environment. Children as small as four or five can be asked to explain the relationships between objects (*How is the bus part of the transportation system? And how can a bus be broken down into smaller parts, like the seats and engine and windows?* Or *how are fruits part of your diet? And what are fruits made of?*). By narrowing in and scoping out, problem-solvers may use *holonic thinking* to change the main focus of the problem, consider the effects of various solutions on the holon, its parts, and those things of which it is also a part, and ultimately to resolve it.

### 6.3. Problems as symbols, order, patterns, categories and relationships: *five pillars of the mind*

*The Five Pillars* refer to the neural networks in the brain related to symbols, patterns, order, relationships and categories (Tokuhama-Espinosa, 2019) and the belief that everything a human can teach or learn has the characteristics of one or more pillars. For example, letters and numbers are symbols; analogical thinking and fractals in nature are patterns; math formulas and sentence patterns are expressions of order; cause and effect in nature as well as the stock market are relationships; parts of speech, types of emotion, and groups of fruits are all categories. The labeling of the five pillars is also interesting, as Tokuhama-Espinosa and Rivera (2013) found that children as young as three-years-old understood what “symbols,” “patterns,” “order,” “relationships,” and “categories” were.

Furthermore, Tokuhama-Espinosa and Rivera discovered that all neuroscientific studies for early math and pre-reading conducted on 0–6 year-olds could be categorized into one of these five pillars, without exception Tokuhama-Espinosa and Rivera (2013). That is, of the nearly 1,000 studies conducted on children at the time (related to math and language), all described neural networks in just these five groupings. This suggested that everything a young child learns related to language and math could be grouped as either a symbol (e.g., letters, numbers, punctuation marks, non-numerical symbols), pattern, order (e.g., sentence structure and grammar, arithmetic equations), relationship (e.g., verb-noun agreement, proportions), and/or category (e.g., word types, positive vs. negative numbers). After this initial study, the authors expanded the inquiry beyond 0–6 years-old and found that research on adult brains could also be grouped into the five pillars.

This mental framework can help in problem-solving when there are many unknowns. That is, sometimes people have problems, and they fail to understand the problem’s origins. Perhaps this occurs because of narrow-band thinking, which seeks out one’s best guess rather than looking for all the evidence (or considering transdisciplinary or holonic thinking). By remembering to identify the symbols, patterns, order, relationships or categories surrounding the problem, the learner may see what was before invisible and embrace the confidence to tackle the problem. For example, if a child has trouble resolving a math problem, teachers can ask them to label all symbols, then ask if they have a problem with any of them. If the problem is not due to symbols, could it be based on patterns (configurations, series, rules or regularity), the order of operations or sequences, categories or the way equivalencies are expressed, or relationships such as an understanding of the core notions of magnitude, or trouble estimating quantities? Using the five pillars as a check list can make it easier to get to the heart of the problem, which then leads to a more accurate intervention and problem resolution.

Whereas the Five Pillars are useful for reminding the learner about what he or she might not be taking into consideration when problem-solving, *Meaning Making* is a way to center the learner’s experience on—and connect them to—the problem and a possible solution (Bornemann and Christen, 2020).

### 6.4. Sense making in problem-solving: *meaning making*

Meaning making is the process through which learners construct understanding from their own personal experiences and the information they encounter (McTighe and Silver, 2020). It is an aspect of human cognition that enables individuals to make sense of the world and confront information. Not only can it be nurtured in schools, but research also suggests this may be of particular importance for adolescents’ developing brains regardless of context (Immordino-Yang and Knecht, 2020; Gotlieb et al., 2022). Evidence suggests that during adolescence, more efficient communication between brain regions supports a surge in higher-level cognitive abilities, which encourages personal, cultural, and emotional meaning-making (Immordino-Yang and Knecht, 2020).

Neuroimaging adds to our understanding of how students make meaning by identifying distinct combinations of neural networks that are employed, as the individual recalls autobiographical information (Sotgiu and Sotgiu, 2021), contrasting it with new information (Ruthven, 2019) that may be charged with emotions (Immordino-Yang and Yang, 2017). fMRI studies of adolescents show coordinated activation of specific neural networks (default mode and salience networks) when individuals watch stories that are emotionally meaningful and personally relevant (Immordino-Yang and Knecht, 2020). This finding suggests that individuals make meaning through both cognitive and emotional approaches, together.

Schools can support meaning making through problem-based learning that leverages student interest, inviting a wider range of concepts, skills, and questions that are personally relevant (Immordino-Yang and Knecht, 2020). Educational practices can support dispositions of mind that encourage the development of meaning-making skills (Immordino-Yang and Knecht, 2020). Overall, meaning making provides an effective mental framework for problem-solving by encouraging reflection, metacognition, and an adaptive, flexible approach, which supports individuals in generating more effective, innovative solutions to novel and complex problems.

As a mental framework, meaning making is an active, reflective process of sensemaking, that simultaneously draws from prior knowledge, emotions, and experiences to construct insights and meaning (Küçüktaş and St Jacques, 2022). Because meaning making involves a high level of reflection and metacognition, it may be used in problem-solving to identify gaps in understanding of others’ thinking or feeling or of one’s own (Jordan, 2011), and to innovate. Perhaps this encourages a more holistic approach to problem-solving, where students learn to consider multiple perspectives (and a wider range of them) in developing comprehensive solutions to problems.

*Transdisciplinary Thinking*, *Holonic Thinking*, the *Five Pillars*, and *Meaning Making* are newer mental frameworks that may be employed by teachers to increase the tools in students’ problem solving, adaptive toolboxes. Along with those shared from education and psychology, they offer the ability to resolve almost any problem one might encounter. We end this section by acknowledging the incomplete nature of the Theory of Frameworks which has yet to be placed within a practical Taxonomy that might facilitate its use.

## 7. The theory of mental frameworks: a taxonomy for problem-solving?

**Figure 1:**
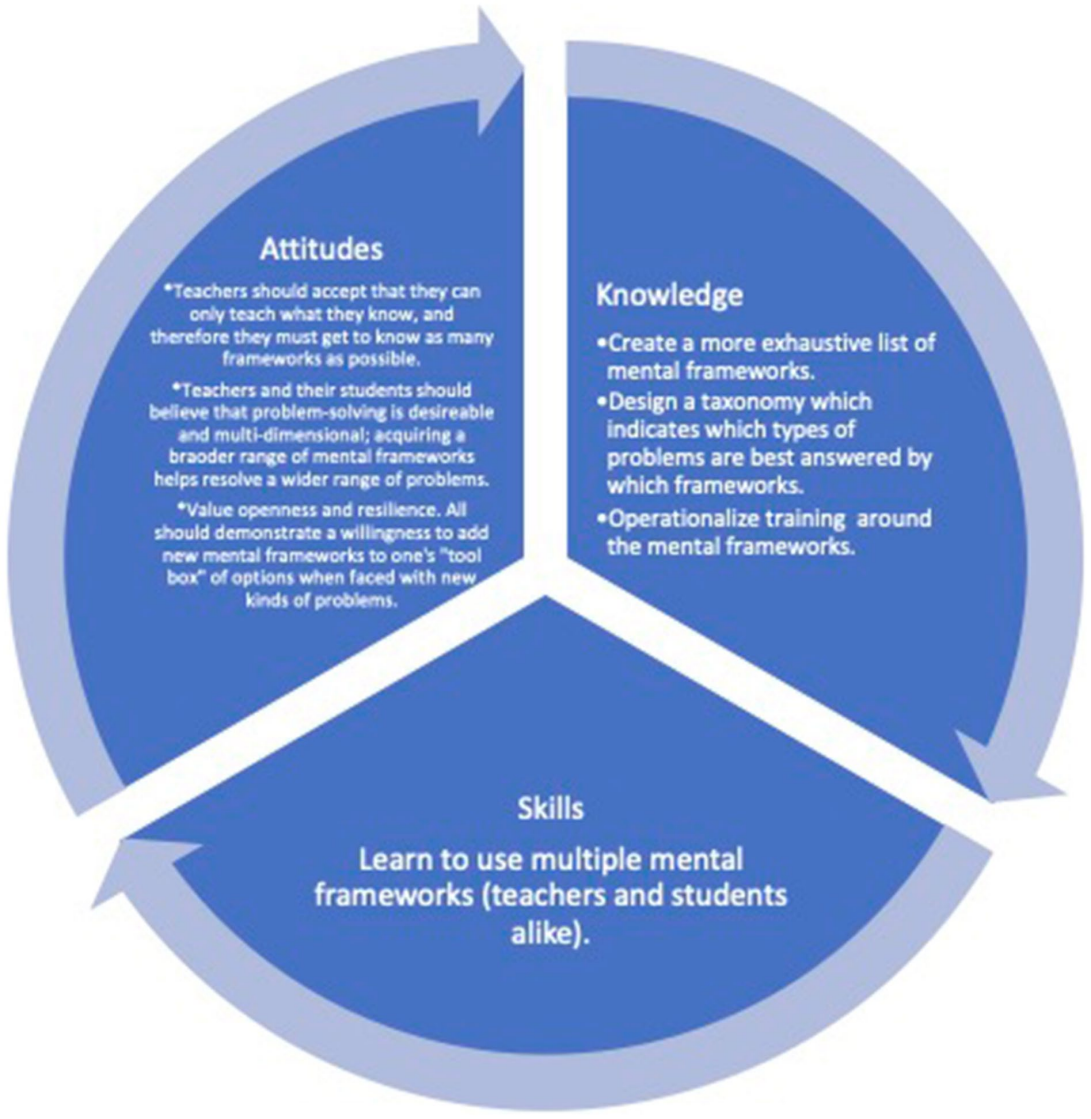
Steps to move from theory to practice.

We propose that the *Theory of Mental Frameworks* would best be expressed as a systems theory, an attempt at addressing and perhaps guiding the complex adaptive system that is the embodied human mind. One tool used in systems theories is that of a taxonomy. Building a taxonomy to organize the mental frameworks is an ongoing process and is beyond the scope of this paper. To further develop this Theory, we will need to generate core competencies, otherwise known as the combination of knowledge, skills and attitudes (OECD, 1997). These will allow us to structure the information in a way that makes the *Theory of Mental Frameworks* practically applicable to all teachers. Some of the competencies needed are summarized in Figure 1.

We acknowledge that ideally, such a tool would capture “all” mental frameworks from education, psychology and neuroscience— a welcome resource for teachers, as taxonomies often serve to succinctly organize knowledge about particular domains and establish common understandings among peers (Unterkalmsteiner and Adbeen, 2023). Perhaps this theory would be best represented as a cyclical taxonomy in which users could select from multiple mental frameworks based on problem-solving needs. Unlike Bloom’s Taxonomy, the content of the mental frameworks are not single words or concepts, which invites speculation as to whether an ontology—a related but distinct approach to classification—might make more sense. Ontology is a set of concepts and categories in a subject area or domain that shows their properties and the relations between them (Oxford, 2023). This is an on-going process for the authors, which we acknowledge renders the explanation in this paper somewhat incomplete, as we believe the goal of a new theory should be in part to create useable knowledge (Connell et al., 2012).

## 8. Discussion

The factors that positively influence human well-being, resilience, and therefore one’s ability to learn and solve problems, informed our process for contemplating which problem-solving frameworks from certain silos to include in the Theory. Specifically, Masten’s research on resilience—which is itself a transdisciplinary area of study—was influential (e.g., Masten, 2001, 2011, 2019). She called resilience “ordinary magic,” which is perhaps also an apt description for mental frameworks at work in problem-solving Masten (2001). It is indeed magical to witness a student encounter dissonance, cogitate, determine where they need support or additional knowledge, and then through a moment of effortful thinking or insight, sail through a complicated or wicked problem to a viable solution, and it often happens below the level of conscious awareness (Stuyck et al., 2022). And though it is magical, it is not rare.

It is our goal through this Theory, at least in part, to make this process visible and teachable. So, if resilience in the face of adversity or stress is defined as one’s ability to positively adapt, to “recover,” “sustain oneself,” or “beat the odds” (Masten, 2001), then perhaps the science of resilience also holds lessons for how to teach the science of thinking and problem-solving, and this deserves more attention beyond the scope of this inquiry. Importantly, studying resilience, and ostensibly problem-solving, through a developmental lens “..may identify windows of opportunity when there is greater plasticity and leverage for change, so that interventions can be effectively tailored and timed for efficacy, adapted to individual, developmental and situational differences” (Masten, 2019, p. 102), an important consideration for applying the *Theory of Mental Frameworks* at different ages and stages during a child’s schooling, and when faced with different kinds of problems.

Because the authors have adopted the medical oath to “do no harm” in this work, and are heavily guided by this value, it must be noted that the *Theory of Mental Frameworks* deserves extensive additional scrutiny and testing, and likely has many limitations (Miles, 2004). Though it is built upon evidence from each of the MBE fields, and contemplates historical perspectives, it is an unproven and hypothetical proposal. The authors look forward to engaging in debate with others who research problem-solving in this context. The research leading to this Theory generated at least five important points of discussion.

### 8.1. Teachers cannot teach what they do not yet know

The development of critical thinking and problem-solving skills requires effective teaching of mental frameworks to underpin them. To teach critical thinking for problem-solving effectively, the teacher must be familiar with the various mental frameworks that are involved in the processes (Thomas and Lok, 2015). Contrary to intuition, teaching one mental framework is not enough, as not all are effective or ideal for all problems or people. Unfamiliarity with mental frameworks will handicap teacher instruction of them as one cannot teach what one does not yet know. Therefore, while equipping students with a range of options may improve the likelihood that they can access an appropriate framework in context, doing so may require extensive repetition to execute, and require intensive teacher training.

While teachers know and use many of the models presented here individually, few may have used multiple frameworks in concert or applied them interchangeably (Hmelo-Silver, 2004). Teachers who value the flexibility of multiple models will be able to model this for their students, while teachers who are unaware may place their students at a disadvantage. Having a range of mental frameworks at their disposal may also aid students in their ability to pivot quickly and adopt a new approach if the first framework is unsuccessful. To achieve this, students need to understand the various cognitive processes involved in critical thinking for problem-solving, including the heuristics and biases that may be present in any mental model, which in some scenarios may occur without their awareness. This kind of higher order thinking can only be developed with guidance by someone more knowledgeable (Hamzah et al., 2022). Without a deep understanding of these elements, teachers’ efforts to develop them in their students may be less effective.

What is more, decoupling the problem-solving process from the typical product, or correct answer, involves disrupting complex heuristics which are hard to remediate in the world of high stakes testing (Jones et al., 2003). Teachers who mistakenly equate higher order thinking with test scores may consider time spent on cultivating multiple frameworks for problem-solving in their students unproductive. To ensure that teachers are equipped with the necessary knowledge, skills, and attitudes to effectively teach critical thinking for problem-solving, it is important to prioritize their own knowledge of and familiarity with these frameworks (Thomas and Lok, 2015). This includes not only providing educators with ample opportunities for professional development (Franco and Vieira, 2019; Celik, 2021), but also ensuring they have access to high-quality resources and support. Notably, the development of these thinking skills is an ongoing process, beginning with initial exposure to the ideas, developing into an understanding of how and to what extent the frameworks may be helpful, and eventually using them heuristically as effective complex problem-solving strategies. And as with any new learning, the learning characteristics of the teacher-as-learner will also impact their ability to upskill in this area.

### 8.2. Flexible thinking via executive functions

In order to apply mental frameworks both teachers and students must be agile and willing. Cognitive flexibility is an executive function that enables individuals to adjust their thoughts and behaviors to meet changing situational demands and has been identified as a necessary skill for personal and professional success in the 21st century (Diamond, 2013; Saleh, 2019; Van Laar et al., 2020; González-Pérez and Ramírez-Montoya, 2022). In an educational context, flexible thinking enables learners to transfer knowledge to new situations, adapt to different learning environments, and find novel solutions (Diamond, 2013). Adaptability is considered a facet of flexible thinking, as it enables learners to engage with new contexts and problems in an efficient manner (Barak and Levenberg, 2016). Adaptable, flexible thinkers are able to approach novel and complex problems effectively, in part because they are able to utilize relevant prior knowledge and transfer (or generalize) it to new situations (Bransford et al., 2000).

Cognitive flexibility is a multifaceted construct with varying components that include set-shifting, task-switching (Miyake et al., 2000; Dajani and Uddin, 2015), and cognitive inhibition (Diamond, 2013). Research has demonstrated the importance of flexible thinking in academic contexts (Blair and Razza, 2007; Diamond and Lee, 2011; Diamond, 2013). Influential work by Blair and Razza (2007) identified cognitive flexibility as a significant predictor of early academic achievement in math and reading and found it also predicted later academic achievement in reading, math and science. These findings suggest that flexible thinking is a crucial skill for success in academic domains as well as in daily life. Therefore, it should be developed as an essential element of one’s education. Research has identified several effective strategies for promoting flexible thinking skills in school-age children, Blair and Razza (2007), Diamond and Lee (2011), Diamond (2013) which have the potential to also improve academic outcomes.

Flexible thinking and the efficient use of mental frameworks can support one another reciprocally. The more knowledge and familiarity one has of these models, the more mental flexibility one might demonstrate in considering, selecting, and applying them to suit a particular context. Similarly, greater flexibility in thinking might also help an individual contemplate numerous frameworks from various perspectives and become a heuristic practice unto itself. Flexible thinking is a crucial executive function that enables individuals to adapt to changing situational demands and solve complex problems, and it is embedded in each of the frameworks themselves. Moreover, it is central to the working theory contemplated herein.

### 8.3. Critical thinking and problem based learning

In addition to cognitive dexterity, critical thinking is worthy of attention in this discussion. While many teachers are very familiar with the term, and may have experimented with it, few have experience in habituating mental frameworks. One approach to developing critical thinking and problem-solving skills in students is problem-based learning (PBL). PBL was originally developed in the 1960’s as a way for professors to help medical students who were struggling to retain information for application in clinical practice (Thorndahl and Stentoft, 2020). These students were missing the reasoning skills that more experienced physicians possessed (Hmelo and Cote, 1996), so PBL was created as a way to support ongoing learning in professional practice (Boud and Feletti, 1997). A scoping review by Thorndahl and Stentoft (2020) found that PBL rapidly spread through higher education in the U.S. and Europe, with numerous universities promoting it to enhance critical thinking skills. The researchers explain that critical thinking and PBL are therefore closely intertwined and are supported by the efficient use of mental frameworks.

While there are numerous applications of PBL, at its core, it involves students working collaboratively to solve or answer complex problems and questions, using their prior knowledge and developing new understandings in the process (Kokotsaki et al., 2016). Through engagement with authentic problems and challenges, students are encouraged to analyze, evaluate, and synthesize information to generate and test possible solutions (Ahlam and Gaber, 2014). This process not only helps students to develop critical thinking skills, but also enhances their ability to transfer these skills to new situations (Savery, 2006). However, as critical thinking is not an innate ability, but rather a set of skills that is developed over time, it is important that educators support students directly in cultivating these skills (Savery, 2006). Teachers can support PBL with explicit instruction of strategies to approach problem solving, including a variety of mental frameworks that will serve students in and beyond the classroom.

### 8.4. Novice to expert

A fourth reflection considers the relationship between mental frameworks and one’s stage as problem-solver (novice to expert). The Dreyfus Model of Skill Acquisition outlined a series of stages through which a learner passes as they go from a beginner or novice, originally knowing nothing about the material or skills at hand, to becoming an expert (e.g., novice, advanced beginner, competent, proficient, and expert; Dreyfus and Dreyfus, 1980; Peña, 2010). Ostensibly, as a person practices they become more competent and along with this competence comes the ability to change and manipulate processes or concepts—they can even be more cognitively flexible and creative (Peskin and Ellenbogen, 2019; Teng et al., 2022). Later adapted by Benner (1982) into the *Novice to Expert* model, it was used for nurse practitioner training. The idea has subsequently been applied outside of medical training spaces and the broader concept is underscored by findings in neuroscience related to neuroplasticity, and how the brain moves from relying on a heavy cognitive consumption when learning something new, to a lower cognitive load as something learned becomes practiced and eventually automated (e.g., Pezzulo et al., 2010; Debarnot et al., 2014; Peskin and Ellenbogen, 2019). Again, we see the important role of heuristics emerge.

In education, a learner progresses from novice to mastery ability, then gains the agility to apply the newly adopted skills, thereby changing their approach to future learning as the process progresses, in what might be described as an upward spiral of learning (e.g., Baynouna Al Ketbi, 2018; Teng et al., 2022). Novice problem-solvers are likely to have fewer strategies for tackling challenges, whereas experts may flow freely and flexibly between approaches.

Learning new mental frameworks might therefore require more effort for novices before becoming effortless or automatic, and teachers can learn to coach students through the stages with patience and persistence, to the benefit of greater learning. Practicing new mental models will support future learning for students who will be experts in their ability to pull from a wider range of thought and problem-solving modes, eventually.

### 8.5. Frameworks alone are not enough

Finally, while having access to mental frameworks will benefit learners by growing their toolbox of options, tools and frameworks alone are not enough. In most senses, less is not more in the world of learning. Indeed, there is much evidence across fields of learning science supporting the idea that more is better in education—more tools, exposure, experiences, practice, channels of delivery, perspectives, skills, knowledge, and of course, mental frameworks. They each contribute to improved mental agility and innovation. As professor of neuroscience, Klemm (2012) reinforced, “the more you know, the more you can know,” and the more mechanisms one will have for tackling more complex problems and solving new ones in the future (Batchelor et al., 2021). What there is not more of, however, is time. It is ironic that problem-solving around the role and nature of schools in society points to the formation of problem solvers themselves, and that implementing a tool that may facilitate this, such as the Theory of Mental Frameworks, requires time to learn.

Currently, many schools find it necessary to prioritize what can easily be measured (Tokuhama-Espinosa, 2014). Straight-forward, quantifiable multiple-choice tests require less time than tracking the development of each child’s mental frameworks. Operationalizing the *Theory of Mental Frameworks* has the potential to meaningfully improve how we teach critical thinking and problem-solving for all types of learners in all types of contexts, as it leverages neuroplasticity to curate vital heuristics that support everything from emotional and cognitive dexterity to executive functions, meaning making, transdisciplinary and holonic thinking, and ultimately, the ability to address wicked problems. But it will take time. This observation suggests that learning about mental frameworks should begin in the earliest school years and be a lifelong pursuit, as problem-solving is a human skill needed at all age levels.

Broadly speaking, we feel teaching based upon the *Theory of Mental Frameworks* will encourage cognitive exploration that: (a) is less linear and predictable in its duration for each student, (b) is less concrete at the outset in determining what the “right” answer(s) to a problem may be, (c) assumes that there are multiple viable approaches and solutions to most problems, and (d) is transferrable to other life contexts. In conclusion, we propose that the *Theory of Mental Frameworks* offers a reliable, transdisciplinary, meta-process for extending adaptive toolkits to approach problems with greater flexibility, adaptability, and with the dexterity to pivot when different approaches are needed.

## Data availability statement

The original contributions presented in the study are included in the article/Supplementary material, further inquiries can be directed to the corresponding author.

## Author contributions

All authors listed have made a substantial, direct, and intellectual contribution to the work and approved it for publication.

## Conflict of interest

Authors TT-E and CB have received payment from Connections: The Learning Sciences Platform for administering teaching courses. Author CB is employed by The Decision Lab.

The remaining authors declare that the research was conducted in the absence of any commercial or financial relationships that could be construed as a potential conflict of interest.

## Publisher’s note

All claims expressed in this article are solely those of the authors and do not necessarily represent those of their affiliated organizations, or those of the publisher, the editors and the reviewers. Any product that may be evaluated in this article, or claim that may be made by its manufacturer, is not guaranteed or endorsed by the publisher.
